# Suppressing Dazl modulates tumorigenicity and stemness in human glioblastoma cells

**DOI:** 10.1186/s12885-020-07155-y

**Published:** 2020-07-18

**Authors:** Fengyu Zhang, Ruilai Liu, Haishi Zhang, Cheng Liu, Chunfang Liu, Yuan Lu

**Affiliations:** 1 Department of Laboratory Medicine, Huashan Hospital, Shanghai Medical College, Fudan University, 12 Wulumuqi Road, Jing-an District, Shanghai, 200040 China; 2 Department of Laboratory Medicine, Shanghai General Hospital, Shanghai Jiao Tong University, 85 Wujin Road, Hongkou District, Shanghai, 200080 China; 3 Department of Neurosurgery, Huashan Hospital, Fudan University, 12 Wulumuqi Road, Jing-an District, Shanghai, 200040 China

**Keywords:** Glioblastoma, Dazl, Cancer-germline, Tumorigenicity, Stemness

## Abstract

**Background:**

Glioblastoma is devastating cancer with a high frequency of occurrence and poor survival rate and it is urgent to discover novel glioblastoma-specific antigens for the therapy. Cancer-germline genes are known to be related to the formation and progression of several cancer types by promoting tumor transformation. Dazl is one such germline gene and is up-regulated in a few germ cell cancers. In this study, we analyzed the expression of Dazl in human glioblastoma tissues and cells, and investigated its significance in proliferation, migration, invasion and chemoresistance of the glioblastoma cell lines.

**Methods:**

We evaluated the expression of Dazl in different pathologic grades of glioblastoma tissues by immunohistochemistry. We assessed the expression of Dazl in glioblastoma cells and normal human astrocytes (NHA) cells by western blotting and RT-qPCR. Then we generated Dazl knockout glioblastoma cell lines using the CRISPR/Cas9 gene-editing technology to explore the cellular function of Dazl. We detected the proliferation and germline traits via CCK-8 assays and alkaline phosphatase staining, respectively. Boyden chamber assays were performed to measure glioblastoma cell migration and invasion. Crystal violet staining was used to determine the number of viable cells after the treatment of Doxorubicin and Temozolomide. Finally, we used subcutaneous xenograft studies to measure the growth of tumors in vivo.

**Results:**

We found that Dazl was upregulated in glioblastoma tissues and glioblastoma cell lines. Dazl knockdown glioblastoma cells showed decreased cellular proliferation, migration, invasion, and resistance in vitro, and inhibited the initiation of glioblastoma in vivo. The glioblastoma cell lines A172, U251, and LN229 were found to express stem cell markers CD133, Oct4, Nanog, and Sox2. The expression of these markers was downregulated in Dazl-deficient cells.

**Conclusions:**

Our results indicated that Dazl contributes to the tumorigenicity of glioblastoma via reducing cell stemness. Therefore, cancer-germline genes might represent a new paradigm of glioblastoma-initiating cells in the treatment of malignant tumors.

## Background

Glioblastoma is among the most prevalent primary brain tumor, accounting for 15–20% of all intracranial tumors. The median survival time is only 15 months. Among these, glioblastoma is characterized by excessive proliferation, high invasion and high resistance to clinical treatment [1–3]. The current standard treatment for glioblastoma patients involves radical surgical resection followed by adjuvant radiation and chemotherapy, numerous antineoplastic drugs such as Doxorubicin (Dox) and Temozolomide (TMZ), are widely used as in clinical treatment of glioblastoma [4, 5]. However, glioblastoma is notorious for its chemoresistance to treatment, and despite many efforts have been made, the addition of Dox and TMZ against glioblastoma have largely failed. Recurrence after chemo- and radiotherapy is inevitable and eventually leads to high mortality in patients with glioblastoma [6]. Tumor initiation, therapeutic resistance, and recurrence originate from cancer-initiating cells (CICs) [7–9]. CICs display some stem cell markers and exhibit sustained self-renewal. Glioblastoma cells with stem characteristics have been isolated from glioblastoma tissues or established glioblastoma cell lines, based on the expression of stem cell markers and the ability to survive in certain stem cell circumstances. Glioblastoma-initiating cells have been found to exhibit resistance to chemotherapy and radiotherapy, tumor-initiating potential, migration, and proliferative capacity [10].

Generally, the concepts of how CICs gain their ability to self-renew and proliferate are hardly understood. In the past decade, Takahashi [11] found that cancer cells could gain the embryonic characteristics enabling self-renew, which might be comparable to the reprogramming of differentiated somatic cells to induced pluripotent stem cells (iPSCs) by introducing embryonic stem cell transcription factors. Meanwhile, cancers acquire characteristic properties by reactivating genes normally expressed in embryonic and fetal life. The description of cancer-embryonic genes like CEA, the anomalous production of human chorionic gonadotrophin by a range of histologically distinct cancers, and the finding that germline genes are involved in the process of invasion and metastases [12, 13]. Previous work focusing on germline traits in cancers led to the discovery of cancer-germline (CG) genes, also called cancer-testis (CT) genes, which are mainly expressed in germline cells and are barely expressed in somatic adult tissues; however, they are abnormally activated in a wide variety of tumors [14]. Some of these human CG genes are suspected to be involved in the germline traits of oncogenesis, such as invasiveness, metastasis, immortality, angiogenesis, and hypomethylation, so they are being studied as biomarkers for cancers [14]. Dazl (*Deleted* in *azoospermia-like*), a member of the DAZ (*Deleted* in *Azoospermia*) gene family, which is also identified as a marker for germ cell identification [15]. Dazl is conserved in all vertebrates and acts as a meiosis-promoting factor in developing germ cells [16]. It is also a “licensing factor” that is required for primordial germ cells (PGCs) sexual differentiation [17]. Dazl can directly regulate apoptosis in PGCs by suppressing the translation of Caspase RNAs, loss of Dazl expression results in apoptosis of the postmigratory germ cells and infertility in both sexes in mice, with germ cell loss during development and a final block at meiosis [18, 19]. During the transition of PGCs into germ cells, Dazl acts as a translational regulator and regulates the transcription of the stemness genes *Sox2*, *Sall4*, and *Suz12* [15, 20]. Sox2 regulates proliferation, migration, invasion, and colony formation of glioblastoma cells [21, 22]. CD133, Oct4, and Nanog are identified as stem/progenitor cell markers of glioblastoma [10] and participate in the tumorigenesis of astrocytic glioblastoma [22–25]. Moreover, Dazl identified as a novel cancer germline gene and could promote the proliferation and resistance to chemical drugs of lung cancer cells by enhancing the translation of RRM2 [26]. However, whether Dazl is involved in the formation of glioblastoma has not been reported. Herein, to explore the correlation of Dazl expression and the tumorigenesis of glioblastoma, we generated glioblastoma Dazl^+/−^ GBM cell lines using the CRISPR/Cas9 gene editing system, and we evaluated that the Dazl knockdown attenuated cell proliferation, reduced cell migration, invasion, and chemo-resistance. These results support the concept that Dazl may be a cancer-germline gene involved in the development of human glioblastoma cells.

## Methods

### Cell culture

Experimental analyses were carried out in vitro using the following cell lines: Normal human astrocytes (NHA) (KG578, KeyGEN, Nanjing, China), A172 and U251 cells (HNC241, HNC1088, FDCC, Shanghai, China), and LN229 cell (the First Affiliated Hospital, Army Medical University). NHA, A172, U251, and LN229 cells were cultured in Dulbecco’s modified Eagle medium (DMEM, HyClone) supplemented with 10% (v/v) fetal bovine serum (FBS, 10270, Life Technologies), 4 mM glutamine, 100 IU/mL penicillin, 100 μg/mL streptomycin and 1% nonessential amino acids (Thermo, Carlsbad, CA, USA). All cell lines were cultured in a 37 °C, 5% CO_2_ incubator and passaged for less than 2 months after thawing.

### CRISPR/Cas9-mediated *Dazl* knockdown

According to the protocol of Ran et al [27], CRISPR/Cas9 gene-editing technology was used to mediate *Dazl* knockdown in GBM cells. To generate Dazl-silenced cells using CRISPR-Cas9 gene-editing technology, two different short guide RNAs (sgRNAs) against DAZL were bought from Sigma (Clone ID: HS5000028071 and HS5000028072). The Dazl-sgRNAs sequences are: GCTGATGAGGACTGGGTGCTGG; GAAGCTTCTTTGCTAGATATGG. The *Dazl* sgRNAs were cloned into a CRISPR/Cas 9-Puro vector: hU6-gRNA-PGK-Puro-T2A-BFP. GBM cells were transfected with CRISPR plasmids and the lenti-cas9 pSpCas9(BB)-2A-GFP (PX458) plasmid (Addgene plasmid #48138) using X-tremeGENE 9 DNA Transfection Reagent (6,365,787,001, Sigma-Aldrich, USA). Lenti-Cas9 and Dazl sgRNA plasmids were transfected at a ratio of 150 ng to 50 ng per well. Puromycin (60210ES25, Yeasen Biotech, China) and blasticidin (15,205, Sigma-Aldrich, USA) selection were performed followed by the transfection. Positive clones were isolated by a medium gradient dilution method, finally confirmed by sequencing. Then Dazl deletion was further verified by Western blotting using anti-Dazl (ab34139, Abcam, USA).

### Western blotting

GBM cells and tissues were harvested and lysed in RIPA lysis buffer (P0013B, Beyotime, China) supplemented with phenylmethanesulfonyl fluoride (PMSF, 1 mM, ST506, Beyotime, China) cocktails. Proteins (25 μg / well) were separated by 10% sodium dodecyl sulfate-polyacrylamide gel electrophoresis and electro-transferred to a polyvinylidene fluoride membrane (Millipore, Bedford, UK). The membrane was blocked with 5% nonfat milk, blotted with primary and secondary antibodies. The immune reaction was detected with an enhanced chemiluminescence substrate (Thermo, USA) using a chemiluminescence imaging system (Clinx, Shanghai, China). Band density was statistically analyzed with ImageJ software. The antibodies used to detect protein expression are shown above.

### RNA isolation and RT-PCR

Total RNA from GBM cells was collected using the Trizol reagent (15,596,018, Thermo, USA) and RNA quantification was done using a NanoDrop2000 spectrophotometer (Thermo, USA) by detecting absorbance at 260 and 280 nm. Subsequently, reverse- transcription of total RNA (500 ng) was performed using a PrimeScript™ RT reagent kit (RR036, Takara, Japan). Quantitative RT-PCR was performed using SYBR premix (RR820, Takara, Japan) and performed on the ABI 7500 system (Life, USA). mRNA expression was normalized to the average of human GAPDH. All reactions were performed in triplicate, and the RNA level was analyzed via the 2^−ΔΔCt^ method. The primers used for detecting gene expression were human Dazl-F: GGTGTCGGGCGCATGTAAT; human Dazl-R: CTTTGGACACACCAGTTCGAT; human GAPDH-F: TGCACCACCAACTGCTTAGC; human GAPDH-R: GGCATGGACTGTGGTCATGAG.

### Immunohistochemistry

Immunohistochemistry for Dazl was done on paraffin tissue array sections. Slices were deparaffinized by incubating in xylene and rehydrated in an ethanol gradient with decreasing amounts of ethanol until the final wash, which was water. After antigen retrieval in sodium citrate-hydrochloric acid buffer (pH 6.0, C8532, Sigma, USA), subsequent steps were to quench endogenous peroxidase activity with a 3% H_2_O_2_ solution. After blocking the sections with 10% goat serum (ab7481, Abcam, USA) for 1 h, the slides were incubated with monoclonal rabbit anti-Dazl antibodies at 4 °C overnight. Next day remove the slices from 4 °C and rewarming at RT 30 min, then all slides were incubated with HRP secondary antibodies and stained with a DAB kit (ab64238, Abcam, USA) and with hematoxylin solution (MHS1, Sigma, USA). Finally, dehydration was performed in 85, 95, and 100% ethanol and distilled water sequentially.

### Cell proliferation assay

According to the manufacturer’s instructions, GBM cells were all planted with a density of 1 × 10^3^ cells per well in 96-well plates. Following the 7 consecutive days culture, each well was replaced with 100 μl fresh DMEM containing 10 μl CCK-8 solution (CK04, DOJINDO, Japan), and incubated at 37 °C for 2 h. The optical density was measured at 450 nm on a microplate reader (Biotek, USA). Background signal was subtracted, all values were repeated 4 times.

### Alkaline phosphatase staining

GBM WT cells and Dazl-knockdown cells were washed with 100 mM Tris-HCl buffer (pH 8.2). For phosphatase activity reaction, cells were treated with a Vector® Blue Alkaline Phosphatase (Blue AP) Substrate kit (SK5300, Vector Laboratories, USA) according to the manufacturer’s instruction. After staining, randomly selected 10 microscopic fields (200 × magnification) for each treatment and counted stain-positive colonies.

### Cell migration and invasion assay

For cell migration assay, GBM cells (5.0 × 10^4^ cells / well) were seeded into the upper chambers of wells in 24-well plates that had 6.5 mm polycarbonate membranes with an 8 um pore size (3422, Corning, USA). For the cell invasion experiment, Matrigel matrix (354,234, Coring, USA) in DMEM (1:3) was coated into the upper chambers. The DMEM was removed carefully when the Matrigel matrix was solidified 12 h later. A total of 5.0 × 10^4^ cells suspended in serum-free DMEM were seeded into the upper chambers. DMEM with 10% FBS was added to the lower chambers. Twenty-four hours later, cells remaining on the upper surfaces of the membranes were removed, with the others that invaded through the membrane filters being fixed with methanol for 30 min, stained with crystal violet (C1021, Beyotime, China) for 30 min, and photographed.

### In vivo experiments: xenograft model

All animal experiments complied with the “Guide for the Care and Use of Laboratory Animals” of the National Institutes of Health and all animal experiments adhered to the ARRIVE guidelines. To explore whether Dazl is involved in the tumorigenicity of glioblastoma in vivo, Dazl knocked-down cells (1.5 × 10^5^) and GBM WT cells (1.5 × 10^5^) were subcutaneously injected into 4-week-old female BALB/c nude mice (*n* = 6 per group, Shanghai Lab. Animal Research Center, China) in their back. Vernier calipers were used to measure the tumor diameter of nude mice every 6 days to assess tumor growth. Tumor volumes were calculated according to the formula: *V* (mm^3^) = *L* × *W*^2^ / 2 (where *V* is the tumor volume, *L* is the length, and *W* is the width). The survival of the remaining mice was assessed via Kaplan-Meier analysis. The mice were euthanized via CO_2_ at the end of the experiments. Tumors from each mouse were removed, photographed, measured, and weighed, then were used for biochemical (frozen tissue) and histological (paraffin fixed tissue) analyses.

### Statistical analysis

Statistical analysis was carried out by using GraphPad Prism version 6.0 (San Diego, CA, USA). Each figure shows an accurate representation of the error bars. Unless otherwise specified, all experiments were performed at least in triplicate. *P* < 0.05 were considered as statistically significant.

## Results

### Upregulation of Dazl expression in both glioblastoma cell lines and glioblastoma tissues

**Fig. 1:**
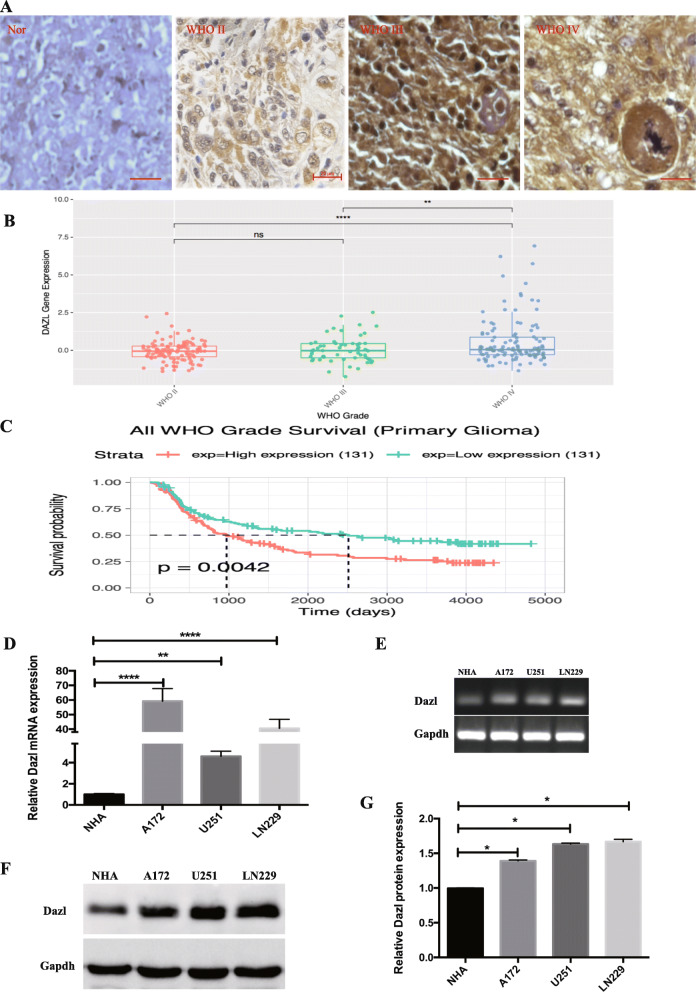
The expression levels of Dazl in glioblastoma tissues and cell lines. **a** Dazl expression was examined by immunohistochemical analysis in human glioblastoma tissues and adjacent normal tissues. Strong cytoplasmic expression of Dazl (brown staining) was detected in stage III/IV glioblastoma cells, and the nucleus was stained blue with hematoxylin. Nor: Normal. Images were taken from the inverted microscope (bars = 50 μm, magnification × 200). **b** The correlation of Dazl expression and glioblastoma grade was analyzed from the Chinese Glioblastoma Genome Atlas (CGGA) data (^**^P < 0.01, ^****^P < 0.001). **c** The correlation of Dazl expression and overall survival of glioblastoma patients was determined from the CGGA data (^**^P < 0.01). **d** The lysates of glioblastoma cells from A172, U251, and LN229 cell lines were harvested and examined for Dazl expression, the cell lysates of NHA cell line were used as the negative control (^**^P < 0.01, ^****^P < 0.001). **e** Detection of gene expression by agarose gel electrophoresis after RT-PCR. **f** Dazl expression in glioblastoma cell lines was detected by western blotting. **g** Relative Dazl expression was quantified by Image J software using Gapdh as an internal control. (^*^P < 0.05)

To determine the clinical significance of the cancer germline gene in glioblastoma, the expression of Dazl was examined by Immunohistochemical (IHC) analysis. Dazl expression was mainly localized in the cytoplasm and detected in the glioblastoma tissue samples with strong staining compared with that in normal brain tissues (*P* < 0.05, Fig. 1a). Furthermore, Dazl expression was increased with the malignant grade of brain glioblastoma based on data from the Chinese Glioblastoma Genome Atlas (CGGA) (*P* < 0.05, Fig. 1b). Dazl was negatively associated with overall patient survival based on the CGGA data (*P* < 0.05, Fig. 1c). We then analyzed the mRNA expression of Dazl in three glioblastoma cell lines and the normal NHA cell lines. High expression of Dazl was evident in A172, U251, and LN229 cells compared to that in NHA cells (*P* < 0.05, Fig.1d and e). Consistent with the mRNA expression, western blotting demonstrated that the protein expression of Dazl in glioblastoma cell lines was significantly increased compared with that in NHA cells (*P* < 0.05, Fig. 1f and g). These results indicated that Dazl is expressed in the glioblastoma cell lines, in line with the observations in glioblastoma tissues.

### Dazl knockdown inhibits the proliferation and germline traits in glioblastoma cells in vitro

**Fig. 2:**
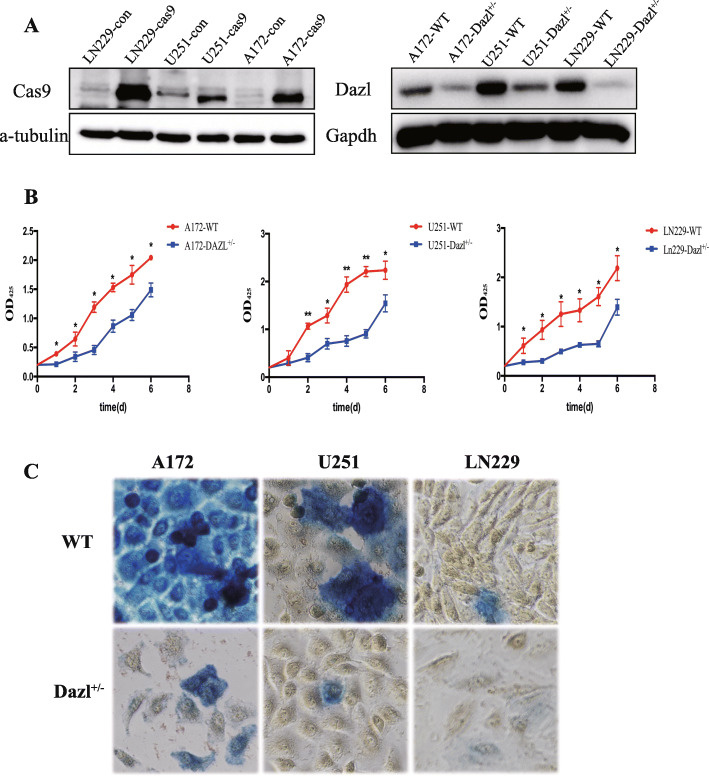
Dazl knockdown inhibited the proliferation and germline traits of glioblastoma cells in vitro. **a** Western blot analysis detected whether Cas9 protein was transfected into GBM cells successfully and whether Dazl protein was deleted. **b** A CCK-8 cell proliferation assay was performed after Dazl deletion in A172, U251, and LN229 cells. **c** An alkaline phosphatase stain assay was performed between the WT GBM cell lines and the Dazl deletion cells. Images were taken from the inverted microscope (magnification × 200). All experiments were carried out in triplicate. Data are shown as the mean ± SE (^*^P < 0.05, ^**^P < 0.01)

To assess the biological functions of Dazl in human glioblastoma, we used the CRISPR/Cas9 system to build Dazl knockdown cell lines. Lenti-Dazl-sgRNA and lenti-Cas9 were co-transfected into A172, U251 and LN229 cell lines, separately, the single colonies from the transfected cells were isolated and analyzed by western blotting. The results showed that glioblastoma cells were successfully transfected with Cas9, and the expression of Dazl protein was inhibited in Dazl knockdown cell lines (*P* < 0.05, Fig. 2a). Since Dazl^−/−^ could completely inhibit the proliferation of glioblastoma cells, all the deletion cell lines we acquired were heterozygous (Dazl^+/−^). Next, we examined whether Dazl is a critical regulator of glioblastoma cell proliferation and detected the effect of Dazl knockdown on glioblastoma cell growth. By knocking down Dazl in A172, U251, and LN229 cell lines, we found that they all displayed decreased proliferation rates compared to that in the Dazl WT cells (*P* < 0.05, Fig. 2b), and the population of cells in sub G1 phase increased significantly, in addition, the cell populations in G2 phase in Dazl KD cells were decreased (Supplement Figure S1). Reduction of Dazl protein levels in A172, U251, and LN229 cell lines reduced colony formation in a soft agar anchorage-independent colony-forming assay (Suppl Figure S2). Furthermore, AP stain showed that Dazl knockdown also reduced the germline characteristics in glioblastoma cells, and germline characteristics might be related to the tumorigenicity of GBM cells (*P* < 0.05, Fig. 2c). These findings demonstrated that Dazl knockdown inhibit the proliferation and germline traits of glioblastoma cell in vitro.

### Knockdown of Dazl inhibits glioblastoma cell migration and invasion in vitro

**Fig. 3:**
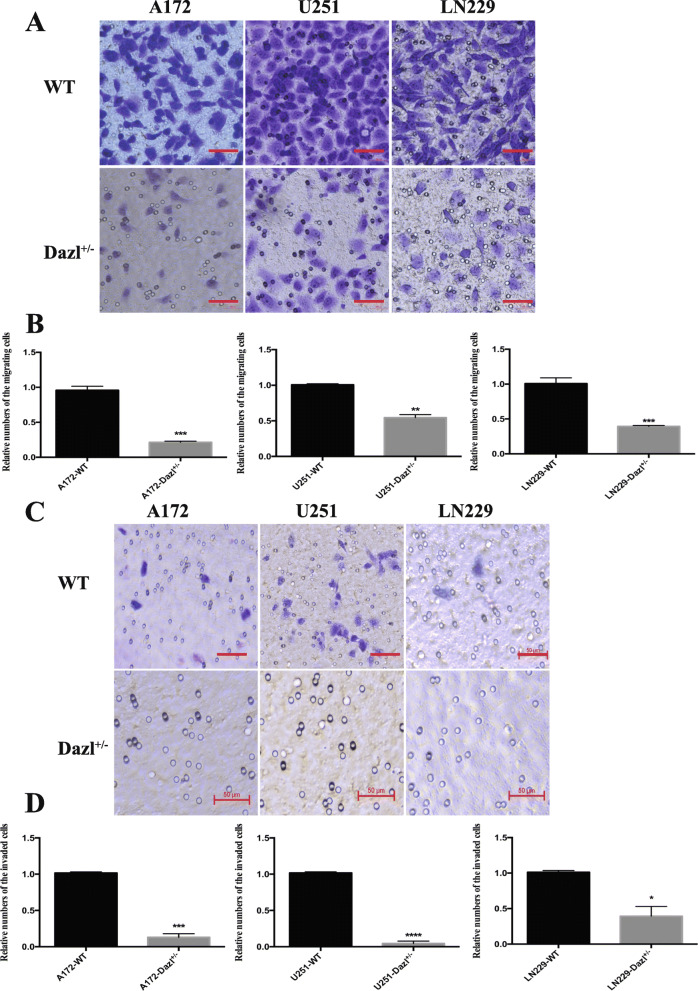
Knockdown of Dazl inhibited glioblastoma cell migration and invasion in vitro. **a** Cell migration assays were performed after Dazl deletion in A172, U251, and LN229 cells. A172, U251, and LN229 cells with Dazl knockdown exhibited decreased ability to migrate through the Boyden chamber compared with the WT GMB cell lines. Five pictures were collected for each group, and the representative images were shown here. Images were taken with an inverted microscope (bars = 50 μm, magnification × 200). **b** The statistical analysis of the ability of the glioblastoma cells’ migration (^**^P < 0.01, ^***^P < 0.001). **c** The invasion of A172, U251, and LN229 cells with Dazl knockdown was measured by transwell assay. Cells migrated through Matrigel-coated transwell inserts and relative invasion proportion of cells were shown. Five pictures were collected for each group, and the representative images were shown here. Images were taken with an inverted microscope (bars = 50 μm, magnification × 200). **d** The statistical analysis of the ability of the glioblastoma cells’ invasion, the numbers of invasion cells with Dazl knockdown were significantly less than those with untreated cells. All experiments were carried out in triplicate. Data are shown as the mean ± SE (^*^P < 0.05, ^***^P < 0.001)

To estimate whether Dazl knockdown affects the migration and invasion of glioblastoma cells. Firstly, we examined cell migration by performing the transwell migration assay. The assay showed that the number of migrated Dazl^+/−^ cells were decreased compared to the Dazl WT cells in migration experiments (*P* < 0.05, Fig. 3a and b). The finding indicated that Dazl deficiency significantly inhibited the migration ability of A172, U251, and LN229 cell lines. Next, we examined the invasion activity by using a Matrigel invasion assay. Cell invasion assays indicated that Dazl knockdown resulted in a significantly lower proportion of cell migration through the Matrigel-coated chamber in contrast to the glioblastoma WT cells (*P* < 0.05, Fig. 3c and d). These results revealed that knockdown of Dazl remarkably inhibited the migration and invasion of glioblastoma cell in vitro.

### Knockdown of Dazl increases the chemosensitivity of glioblastoma cells to DOX and TMZ in vitro

**Fig. 4:**
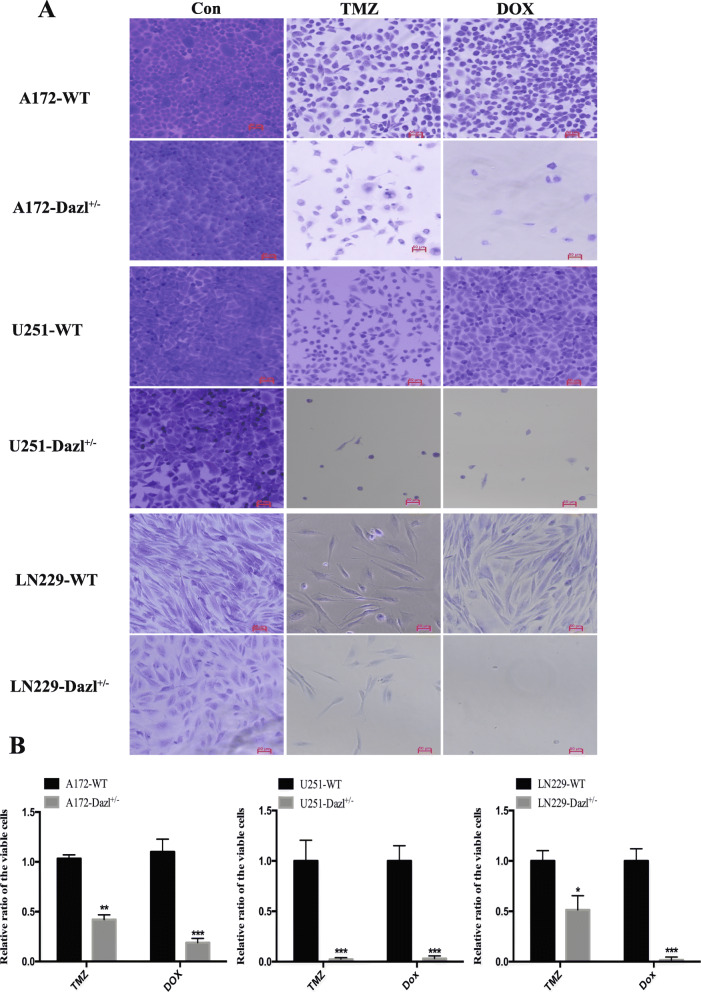
Knockdown of Dazl inhibited the resistance of glioblastoma cells in vitro. **a** Dazl KD cells were significantly more sensitive to TMZ and DOX than GBM WT cells (A172, U251, and LN229). Images were taken with an inverted microscope (bars = 50 μm, magnification × 100). **b** The statistical analysis of the glioblastoma cell resistance. All experiments were carried out in triplicate. Data are shown as the mean ± SE (^*^P < 0.05, ^**^P < 0.01, ^***^P < 0.001)

The role of the Dazl gene in the sensitivity of GBM cells to TMZ and DOX was explored by incubating the GBM cells in which Dazl was knocked down, with TMZ and DOX for 48 h. Under a light microscope, in the presence of TMZ and DOX, the number of A172, U251, and LN229 cells per field of vision showed lower in the Dazl KD group, in contrast to the glioblastoma WT cells (*P* < 0.05, Fig. 4a and b). These results revealed the involvement of Dazl in the sensitivity of glioblastoma cells to TMZ and DOX.

### Dazl inhibits the initiation of glioblastoma via blocking the stemness of glioblastoma cells

**Fig. 5:**
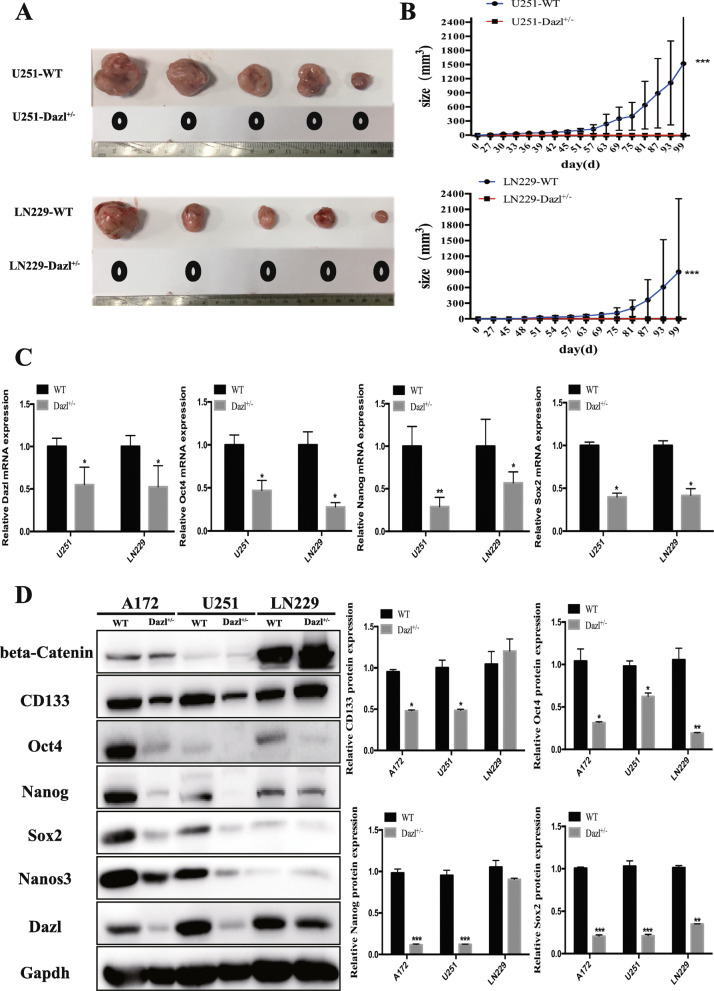
Dazl inhibited the formation of glioblastoma via inhibiting the stemness of glioblastoma cells. **a** Tumor growth was observed from GBM cells with Dazl alterations that were implanted subcutaneously in nude mice, *n* = 5; tumors were excised, photographed, and measured; **b** The tumor growth sizes were recorded every 6 days between Dazl WT and Dazl ^+/−^ GBM cell lines in xenograft tumor models, *n* = 5; data are shown as the mean ± SE. (^***^P < 0.001). **c** Q-RT-PCR analysis of stem cell gene expression in Dazl knockdown GBM cell lines. (^*^P < 0.05, ^**^P < 0.01). **d** Western blot analysis of the relative protein levels of CD133, Oct4, Nanog, and Sox2 in Dazl^+/−^ and WT GBM cells. Quantitative analysis of the relative protein levels of CD133, Oct4, Nanog, and Sox2 in GBM cells was carried out in triplicate. Data are shown as the mean ± SE. (^*^P < 0.05, ^**^P < 0.01, ^***^P < 0.001)

To validate the contribution of Dazl knockdown on glioblastoma tumorigenesis in vivo, we subcutaneously injected GBM WT cells and GBM Dazl^+/−^ cells into the backs of nude mice to build a xenograft model. The growth curve of xenografted tumors displayed that U251 and LN229 cells showed rapid tumor growth in vivo (*P* < 0.05, Fig. 5a and b), whereas U251 Dazl^+/−^ and LN229 Dazl^+/−^ cells markedly inhibited tumor growth. These results suggested that Dazl knocked-down GBM cells were unable to initiate tumorigenesis in 6 months, and recipient mice remained survival after 6 months.

The high post-surgical recurrence rate of glioblastoma is mainly attributed to the existence of cancer stem cells (CSCs) which can promote tumor initiation, invasion, metastasis, and increase both differentiation and proliferation [28, 29]. To support this hypothesis, we then investigated the correlation between Dazl expression and cell stemness. We explored whether the stem transcriptional core formed by Oct4, Nanog, and Sox2 was altered by Dazl levels. By qRT-PCR (*P* < 0.05, Fig. 5c), we found that Dazl knockdown could significantly reduce Oct4, Nanog, and Sox2 mRNA expression. Furthermore, western blot experiments (*P* < 0.05, Fig. 5d) were utilized to detect the protein expression, and Dazl knocked-down glioblastoma cell lines showed significantly reduced expression of stemness markers CD133, Oct4, Nanog, and Sox2, and no changes in the protein expression of beta-Catenin. These results showed that Dazl induces the tumorigenesis in glioblastoma mainly by increasing the stemness but not via the WNT signaling pathway (Fig. 5c and d). Therefore, our reports discovered that germline characteristics of glioblastoma cells were markedly reduced in Dazl knockdown cells, and the germline characteristics might be related to the oncogenicity of glioblastoma.

## Discussion

CRISPR/Cas9 technology is a powerful method for targeting desired genomic sites for gene editing or activity modulation via specific single-guide RNAs (sgRNAs) [30]. In the experiment, *Dazl*-sgRNAs were designed, synthesized and cloned into a lentiviral vector, which was subsequently transduced into glioma cells at a low multiplicity of infection to ensure that only one sgRNA copy was integrated per cell; then, the Cas9 enzyme was guided to the *Dazl* gene location, where Cas9 induced a double-strand break [31] The repair of such a break by glioma cells led to a knockout of the targeted *Dazl* gene, and the Dazl^+/−^ GBM cell lines grew stably for generations. CRISPR knockout technology has been highly effective in identifying genes that have functions in tumorigenesis. CRISPR/Cas9 is the most commonly applied method for generating clinical trials of human cancer [32], and it is far superior to the previously reported RNA interference technology because it ensures the functional stability of the Dazl gene in the cell inheritance.

Glioblastoma is one of the most malignant primary brain tumors associated with poor prognosis and low median survival [33, 34]. Glioblastoma is not a surgically curable disease because the glioblastoma cells invade the surrounding brain tissue and are among the most resistant to chemotherapy [35, 36]. Therefore, new targets in molecular knowledge, prognosis factor, and treatment are urgent. The similarity of the biological characteristics of cancer cells and germ cells prompted Lloyd J. Old to discover cancer/testis (CT) antigens [37]. The discovery elaborated a theory that aberrant expression of germline genes in cancers reflects the activation of the silenced gametogenic programme in somatic cells, and this programmatic acquisition is one of the driving forces of tumorigenesis. Extensive data have been assembled concerning the ectopic activation of germline genes in the progression of several human cancer types [38]. Dazl is responsible for germline traits and plays a central role in controlling pluripotency, differentiation, and apoptosis [15]. In this study, we demonstrated that ectopic expression of the germline gene Dazl in human glioblastoma and its association with tumorgenicity. We found that Dazl promotes cell proliferation in GBM since A172, U251, and LN229 GBM cells with Dazl knockdown exhibited a reduced cell proliferation rate (Fig. 2). We also showed that Dazl increases the ability of migration and invasion through the transwell assays (Fig. 3). Also, TMZ and Dox treated cell lines showed increased apoptosis in A172, U251, and LN229 GBM cells with Dazl knockdown (Fig. 4), which suggested the anti-apoptosis function of Dazl in GBM cells. Lastly, a screening of stem cell markers found that their expression decreased significantly in Dazl-knockdown cells (Fig. 5), suggesting the involvement of Dazl in the maintenance of the glioblastoma stem cell population.

Our work successfully discovered the relationship between Dazl and the proliferation of GBM cells. Dazl has been known for its involvement in cell proliferation in the integrity of PGCs in many vertebrates [39], Dazl is involved in the early proliferation of the germ cells [40], and also have essential roles in controlling a network of cell-cycle regulatory genes such as *sox3* and *Atm* [41]. Dazl enhances postnatal germ cell survival via poly A-proximal interactions that promote the cell-cycle regulation and germ cell survival [41]. Dazl can also improve the spermatogonia proliferation via increasing steady-state levels of inherently unstable mRNA to ensure the high concentrations of regulatory factors in the germ cell development [42]. In this work, we found that Dazl was upregulated in GBM cells and glioblastoma tissues, especially in late-stage. These findings support the oncogenic function of Dazl in tumor formation and proliferation.

Besides regulating cell proliferation, Dazl was also found to be responsible for anti-apoptosis in GBM cells in this work. We found that multiple the number of GBM cells with Dazl knockdown experienced apoptosis compared to normal GBM cells under drug treatment (Fig. 4). Dazl regulates the expression of the key caspases, reveals a meaningful fail-safe mechanism that prevents stray PGCs from forming teratomas by sensitizing them to apoptotic cell death [15, 39, 43]. A previous study also demonstrated that the loss of PGCs in the Dazl^−/−^ embryo is due to increased apoptosis [43] and Dazl knockdown in PGCs causes increased apoptosis. Therefore, the silencing of Dazl induced drug susceptibility in glioblastoma cells by increasing apoptosis. Stemness is thought to be the main reason for chemoresistance, then we detected whether Dazl could regulate the stemness marker in glioblastoma.

Finally, we found that GBM cell lines A172, U251, and LN229 all expressed stemness markers CD133, Nanog, Oct4, and Sox2. Dazl-knocked down cell lines showed significantly decreased expression of these markers. Interestingly, at the transition of PGCs, Dazl-mediated silencing of both pluripotency factors and the polycomb complex allows PGCs to reduce the risk of teratoma formation by inhibition of the pluripotent program while simultaneously preventing somatic differentiation [15]. Dazl likely plays different roles in different developmental stages, and its role in a specific tissue remains the same in both normal and tumor cells. This findings confirm the theory that CG genes could exist in the GBM cells, and mainly present in reproductive tissues, such as testes, fetal ovaries, and trophoblasts, and are aberrantly expressed in a range of human cancers, but have limited expression on other normal tissues in adults [44]. Our results further demonstrated the cancer-promoting role of Dazl in glioblastoma cells and helped expand the knowledge that the germline gene could involve in the formation of glioblastomas. The tumor-suppressive effect of Dazl was exerted through inhibiting the transcriptional activity of Oct4, Sox2, and Nanog gene to attenuate the stemness and resistance of glioblastoma cells. However, our results do not discover the detailed mechanism of the Dazl regulates the tumorigenicity and stemness in glioblastoma cells. Further studies on Dazl expression and its function on these stemness markers should prove beneficial. Moreover, the relationship between germline genes and the tumorigenicity merits further investigation as they are involved in several important cellular signaling pathways. However, our study demonstrated that Dazl promotes the expression of stem cell markers and apoptosis of the GBM cells not through the WNT/beta-Catenin pathway.

## Conclusion

In conclusion, Dazl functions as a novel cancer germline gene to initiate the stemness of glioblastoma cells by regulating the CD133/Oct4/Nanog/Sox2 regulatory axis and increasing the resistance of glioblastoma cells to Dox and TMZ. Additionally, Dazl knockdown not only promotes the glioblastoma cells proliferation, migration, and invasion in vitro, but also inhibits the initiation of glioblastoma in vivo. Therefore, understanding the underlying mechanisms of the cancer-germline gene in glioblastoma has new implications in future therapies to inhibit glioblastoma progression and recurrence.

## Supplementary information

**Additional file 1.**

**Additional file 2.**

**Additional file 3.**

## Data Availability

All data generated or analyzed during this study are included in this published article.

## Abbreviations

AP: Alkaline phosphatase
BFP: Blue fluorescent protein
BSA: bovine serum albumin
CCK-8: Cell counting kit-8
CG: Cancer-germline
CICs: Cancer-initiating cells
CRISPR: Clustered regularly interspaced short palindromic repeats
CSCs: Cancer stem cells
Dazl: *Deleted* in *azoospermia-like*
DMEM: Dulbecco’s modified eagle medium
DOX: Doxorubicin
GBM: Glioblastoma multiforme
NHA: Normal human astrocytes
iPSCs: Induced pluripotent stem cells
KD: Knockdown
PBS: Phosphate-buffered saline
PGCs: Primordial germ cells
RT: Room temperature
sgRNAs: Single guide RNAs
TMZ: Temozolomide
WT: wild type
