# Supplementary material for: Suppressing Dazl modulates tumorigenicity and stemness in human glioblastoma cells
Source: BMC Cancer. 2020 Jul 18;20:673. doi: 10.1186/s12885-020-07155-y (PMC7368788; doi:10.1186/s12885-020-07155-y)
Supplement: Supplementary file 1 — Additional file 1. [file 12885_2020_7155_MOESM1_ESM.docx]

**Additional File 1**: Full western blots of gels from Figure 1F. NHA, A172, U251, LN229, and U118-MG cells were probed with Dazl and Gapdh antibodies. Molecular weight (kDa) markers (MW) are shown: Protein Ladder (Thermo marker, #26616), The molecular weight of Dazl is 37 kDa.





35

40

MW

25

NHA

NHA

A172

LN229

U251

U118-MG

Gapdh





35

40

MW

25

NHA

NHA

A172

U251

U118-MG

LN229

Dazl

Full bands of gels from Figure 1D,E. RT-PCR products were demonstrated by agarose gel electrophoresis. NHA, A172, U251, and LN229 cells were probed with Dazl and Gapdh. Molecular weight (bp) markers are shown: DNA Ladder (TIANGEN, D2000 DNA marker, MD114).


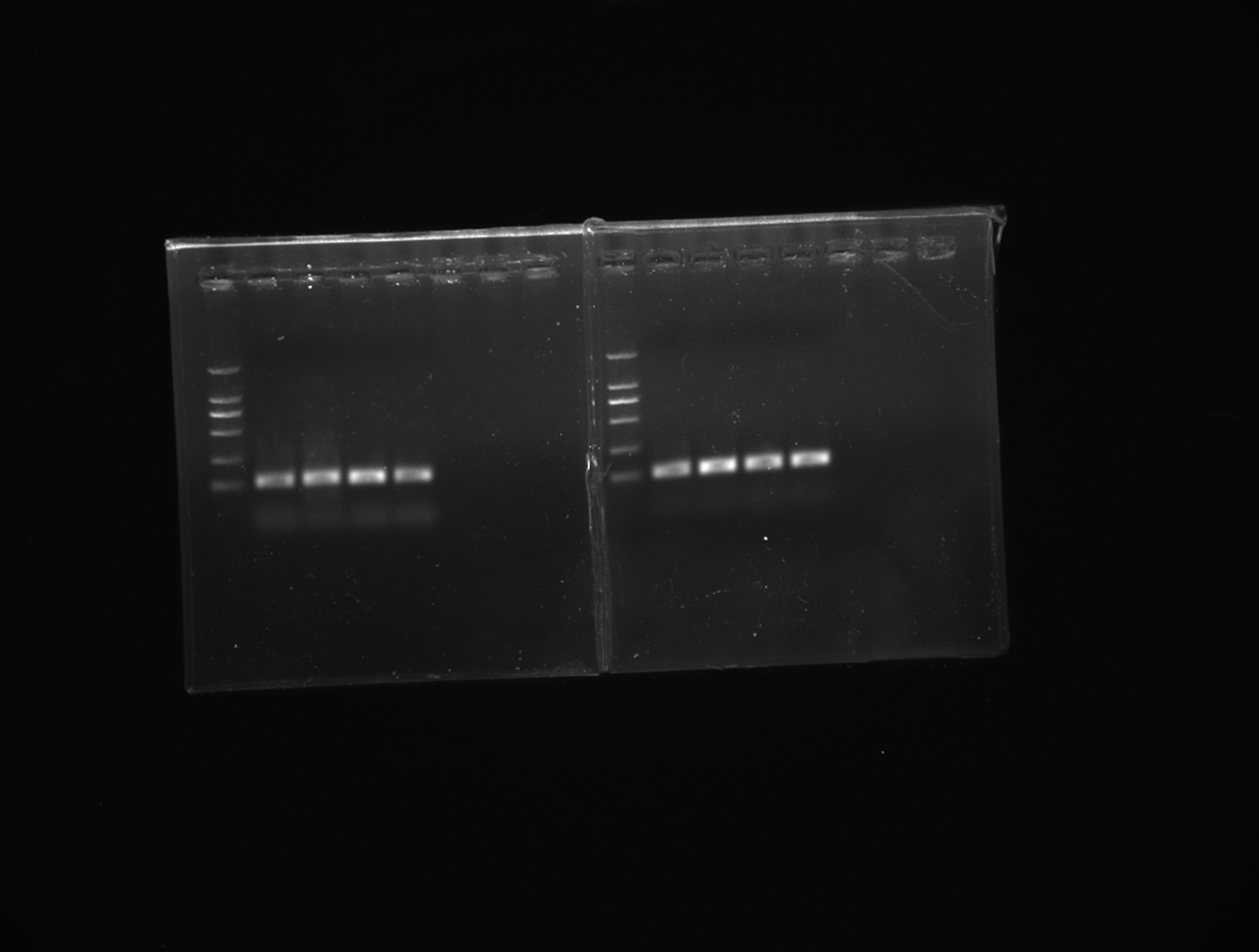


2000

750

bp

1000

250

100

nanos3

Dazl

M

NHA

LN229

A172

U251

M

NHA

A172

U251

LN229


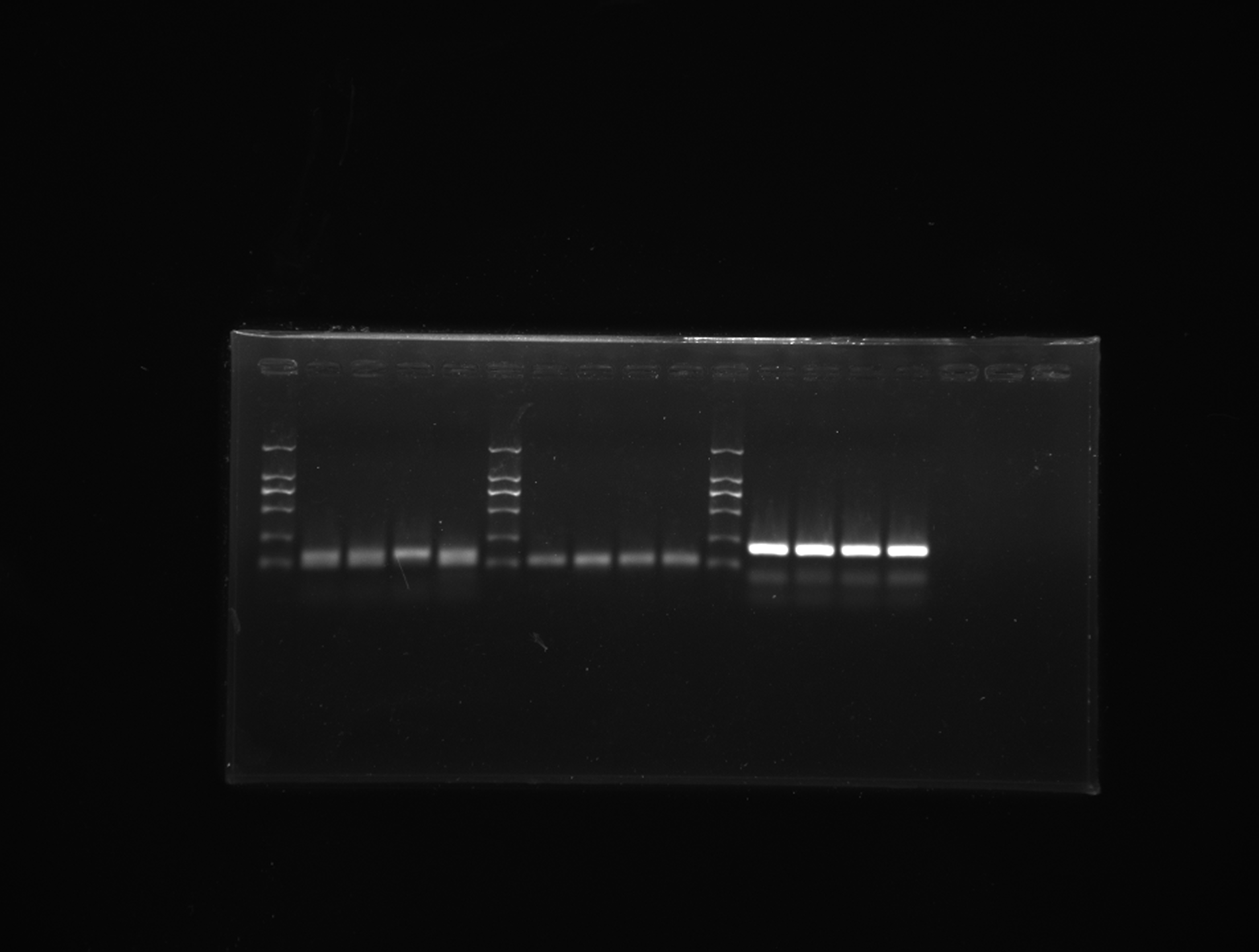


2000

1000

750

500

250

100

M

bp

M

M

gapdh

sox2

nanog

NHA

A172

U251

LN229

NHA

A172

U251

LN229

NHA

A172

U251

LN229
