# Supplementary material for: Suppressing Dazl modulates tumorigenicity and stemness in human glioblastoma cells
Source: BMC Cancer. 2020 Jul 18;20:673. doi: 10.1186/s12885-020-07155-y (PMC7368788; doi:10.1186/s12885-020-07155-y)
Supplement: Supplementary file 2 — Additional file 2. [file 12885_2020_7155_MOESM2_ESM.docx]

**Additional File 2**: Full western blots of gels from Figure 2A. A172, U251, LN229 cells were transfected with cas9,and western blot assay demonstrated the results with cas9 and a-tubulin antibodies. Molecular weight (kDa) markers (MW) are shown: Protein Ladder (Thermo marker, #26616), The molecular weight of cas9 is 158 kDa, a-tubulin is 57kDa.

Full western blots of gels from Figure 2B. Western blot analysis detected whether Dazl protein was deleted. western blot assay demonstrated the results with Dazl and gapdh antibodies. Molecular weight (kDa) markers (MW) are shown: Protein Ladder (Thermo marker, #26616), The molecular weight of Dazl is 37 kDa, Gapdh is 36kDa.
