# Supplementary material for: Suppressing Dazl modulates tumorigenicity and stemness in human glioblastoma cells
Source: BMC Cancer. 2020 Jul 18;20:673. doi: 10.1186/s12885-020-07155-y (PMC7368788; doi:10.1186/s12885-020-07155-y)

**Additional File 3:** Full western blots of gels from Figure 5. Western blot analysis of the relative protein levels of CD133, Oct4, Nanog, and Sox2 in Dazl+/- and WT GBM cells. Quantitative analysis of the relative protein levels of CD133, Oct4, Nanog, and Sox2 in GBM cells was carried out in triplicate. NHA, A172, U251, LN229 cells were probed with beta-Catenin, CD133, Oct4, Nanog, Sox2, Nanos3, Dazl and Gapdh antibodies. Molecular weight (kDa) markers (MW) are shown: Protein Ladder (Thermo marker, #26616), The molecular weight of b-catenin is 92 kDa, CD133 is 120kDa, Oct4 is 43kDa, Nanog is 37kDa, Sox2 is 40kDa, Nanos3 is19kDa, Dazl is 37kDa, Gapdh is 36kDa.

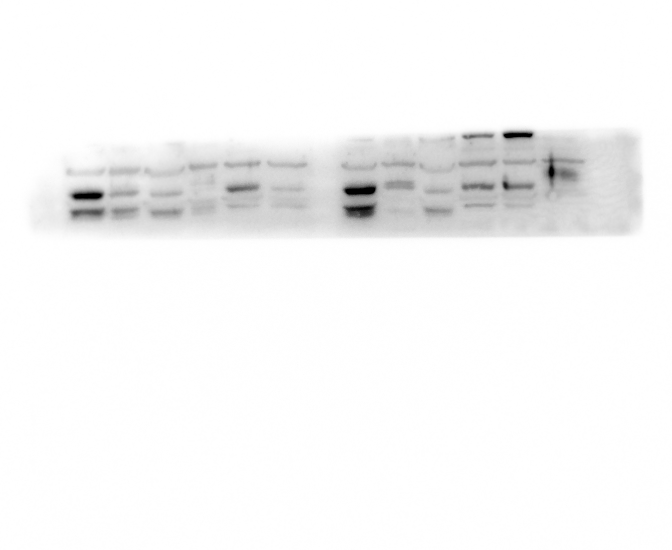

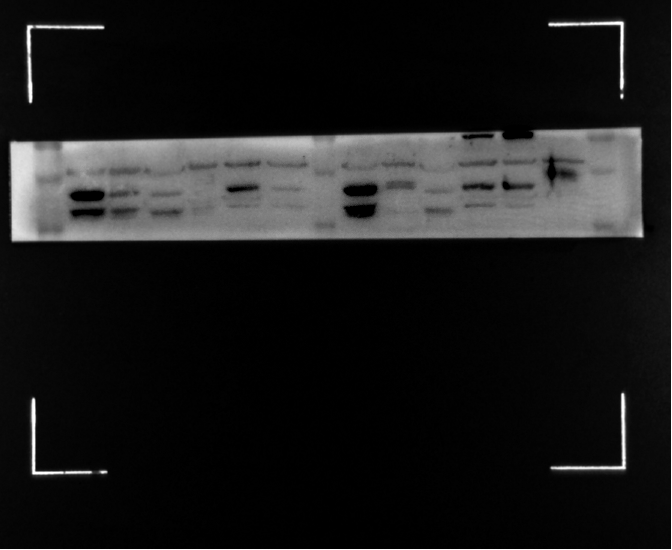


Oct4


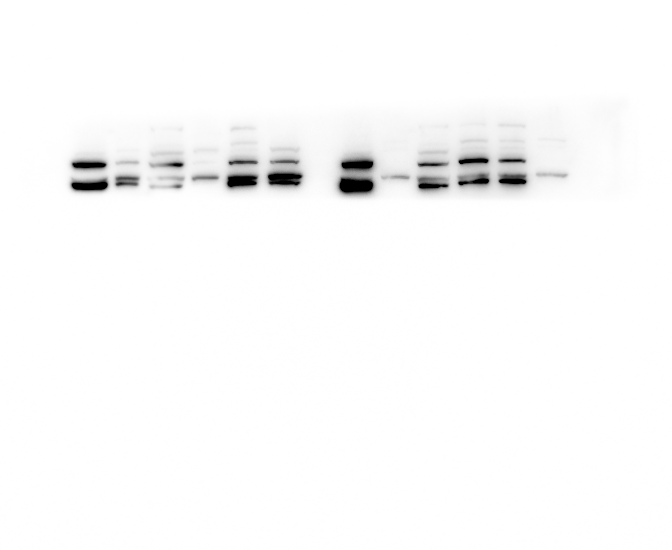

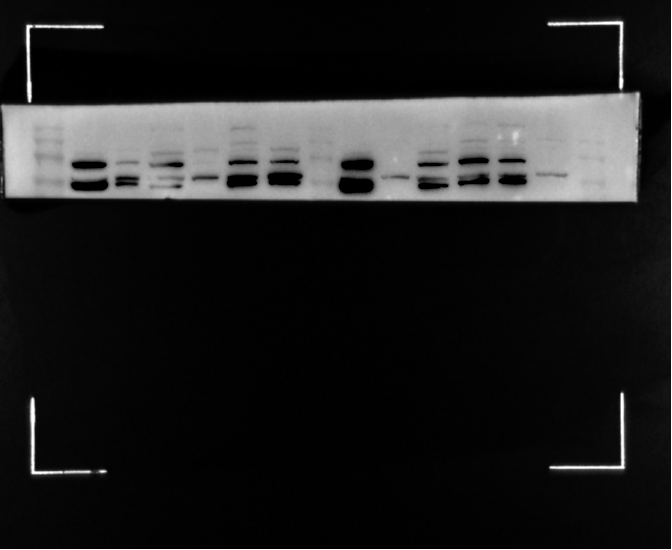


Nanog


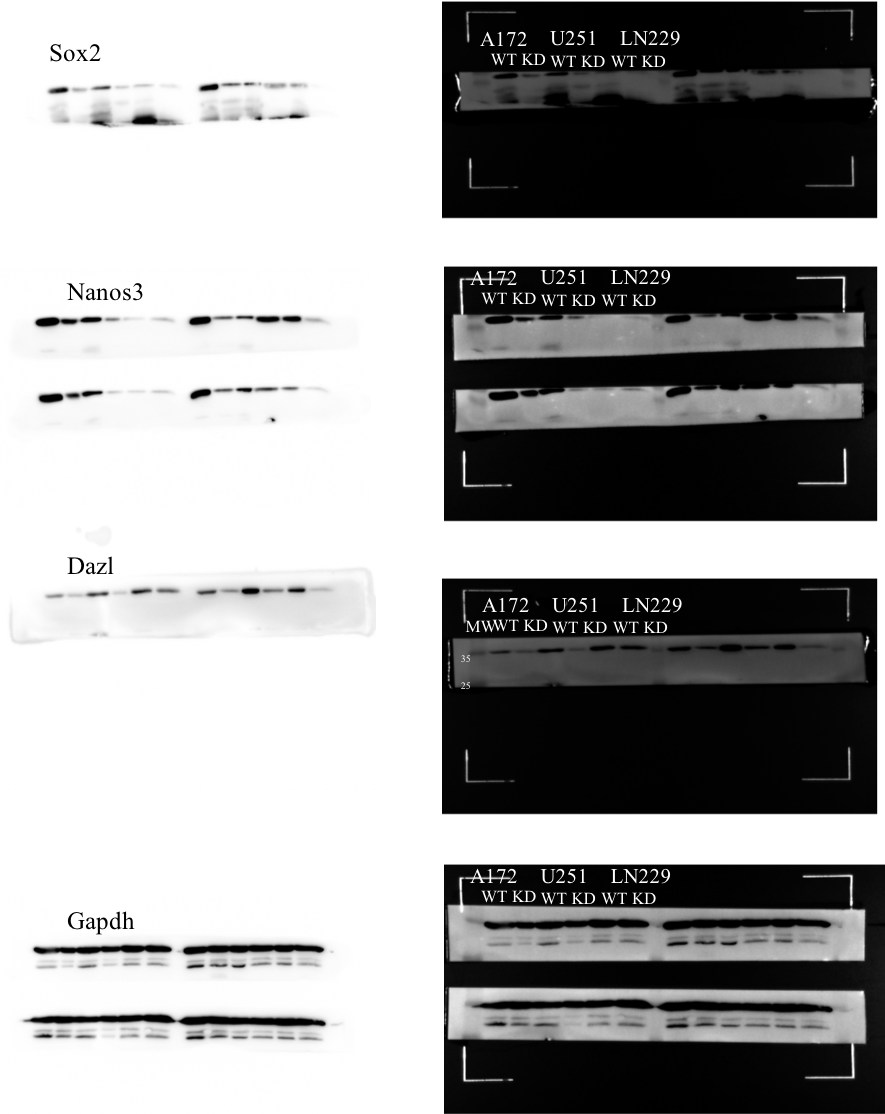

Supplement: Supplementary file 3 — Additional file 3. [file 12885_2020_7155_MOESM3_ESM.docx]
